# Immobilization of Phospholipase A1 Using a Protein-Inorganic Hybrid System

**DOI:** 10.3390/polym13172865

**Published:** 2021-08-26

**Authors:** Shi Cheng, Zitao Guo, Chaojuan Liang, Yi Shi, Peng Geng, Yu Xin, Zhenghua Gu, Liang Zhang

**Affiliations:** National Engineering Laboratory for Cereal Fermentation Technology, Jiangnan University, Wuxi 214122, China; jack1181@sina.com (S.C.); guozt@jiangnan.edu.cn (Z.G.); L1229469055@outlook.com (C.L.); shiyi0621@jiangnan.edu.cn (Y.S.); gengp@outlook.com (P.G.); yuxin@jiangnan.edu.cn (Y.X.); guzhenghua2021@yeah.net (Z.G.)

**Keywords:** phospholipase A1, enzymatic properties, metalloenzyme hybridization, nanostructures

## Abstract

In this study, four kinds of phospholipase A1-metal (Al/Co/Cu/Mn) hybrid nanostructures were prepared for enhancing the stability of the free PLA1. The formed hybrid complexes were characterized by scanning electron microscope (SEM), Fourier infrared spectroscopy (FTIR), and *X*-ray diffraction (XRD). The stability and substrate specificity of immobilized enzymes were subsequently determined. After immobilization, the temperature tolerance of PLA1–metal hybrid nanostructures was enhanced. The relative activity of PLA1–Al/Co/Cu hybrid nanostructures remained above 60% at 50 °C, while that of free enzyme was below 5%. The thermal transition temperature measured by differential scanning calorimetry (DSC) was found to increase from 65.59 °C (free enzyme) to 173.14 °C, 123.67 °C, 96.31 °C, and 114.79 °C, referring to PLA1–Cu/Co/Al/Mn hybrid nanostructures, respectively. Additionally, after a storage for fourteen days at 4 °C, the immobilized enzymes could exhibit approximately 60% of the initial activity, while the free PLA1 was inactivated after four days of storage. In brief, using Co^2+^, Cu^2+^, Al^3+^, and Mn^2+^ as the hybridization materials for immobilization could improve the catalytic properties and stability of the free PLA1, suggesting a promising method for a wider application of PLA1 in many fields such as food, cosmetics, and the pharmaceutical industry.

## 1. Introduction

Phospholipase is a kind of enzyme that can specifically hydrolyze glycerophospholipids at different ester bonds. Based on the difference hydrolysis sites, phospholipases are mainly classified into five categories, referring to phospholipase A_1_ (PLA_1_), phospholipase A_2_ (PLA_2_), phospholipase B (PLB), phospholipase C (PLC), and phospholipases D (PLD) [1]. In nature, phospholipases are widely distributed and have been found in bee venom, snake venom, animal pancreas, plant tissues, and some *Streptomyces*. They could exert toxic and pharmacological effects, such as neurotoxicity, hemolysis, edema, pro-inflammatory, platelet aggregation inhibition, and anticoagulation [2,3,4]. Moreover, phospholipase plays a certain physiological function, including the digestion and decomposition of extracellular phospholipids, participation in the maintenance and remodeling of cell membrane, and the generation of small lipid molecules with cell signal transduction function. At present, phospholipases from microorganisms have been attracting more attention due to the advantage that their production could be mass prepared via fermentation. Currently, the microorganisms that have been reported with the capability to secrete phospholipases include *Candida albicans* [5], *Lactobacillus casei* [6], *Serratia marcescens* [7], *Saccharomyces cerevisiae* [8,9], and *Streptomyces* [10]. PLA_1_ (EC 3.1.1.32) has been verified to show specific hydrolyzing ability against the sn-1 acyl group of phospholipids with the production of lysophosphatidylcholine and a single fatty acid. Relying on its catalytic property, PLA_1_ plays an important role in the chemical industry involving the application or production of fat and oil [11]. Fields such as oil degumming [12], baked foods [13], dairy products [14], and egg yolk processing [15] have wildly introduced the utilization of PLA_1_. In addition, the lysophospholipids obtained from the hydrolysis of PLA_1_ are used extensively in food, cosmetics, and pharmaceutical industries as well [16]. However, a further industrial application of free PLA_1_ is limited to some extent by its relative high price, poor stability, and the difficulty in separation and recovery from the reaction system.

Enzyme immobilization is a technology that could limit the free enzyme in a certain space or have the enzyme completely adhere to a solid structure without movement [17,18]. It is a common, effective, and convenient means of biological enzyme modification, which can greatly improve the catalytic activity and stability of the enzyme [19]. At present, immobilization technology is mainly classified into four categories: adsorption, covalent combination, embedding, and crosslinking [20]. For phospholipase, several strategies have been introduced whilst none of these methods could meet the entire immobilization requirements for PLA_1_. For example, Chen et al. [21] used natural polymer materials to immobilize phospholipase, leading to high mass transfer efficiency. However, polymer materials tended to dissolve under the condition of low mechanical strength. Li et al. [22] immobilized PLA_1_ on magnetic nanoparticle carriers, which were complicated to prepare and required large quantities of enzymes. Li et al. [23] employed an ion exchange resin to adsorb and immobilize phospholipases. However, with the increase of reaction times, the substrate would accumulate with the product, and the mass transfer efficiency would then decrease. 

Organic–inorganic hybrid nanostructures are a kind of immobilization technology that have enzyme and inorganic components assembled with a flower-like three-dimensional hierarchical nanostructure. The nanostructures crystal structure formed by this immobilization method not only has a high specific surface area, but also improves the stability of enzymes due to the formation of stable hybrid crystal structure. Moreover, the metal ions in the hybrid nanostructures could also enhance the activity of some enzymes, and even have the immobilized enzyme catalytic activity greater than that of the free enzyme. Since the first report of protein–inorganic hybrid nanostructures in 2012 [24,25,26], this method had been successfully employed to immobilize many types of enzymes (i.e., laccase, lipase, carbohydrase, and cholesterol oxidase) [27,28,29,30,31]. For developing hybrid nanostructures, Cu^2+^, Ca^2+^, Mn^2+^, Mg^2+^, Zn^2+^, Co^2+^, and Fe^2+^ are the mainly inorganic components that have been reported [32,33,34,35]. In general, depending on the high stability, durability, reusability, and biocompatibility, these protein–inorganic hybrid nanostructures show an extensive utilization in many fields such as biosensor manufacturing, food processing, and pharmaceuticals producing. 

In this study, a recombinant PLA_1_-producing *E. coli* kept in our laboratory was applied to express PLA_1_, and the free PLA_1_ was obtained after purification using affinity chromatography [36]. According to our unpublished study of enzyme properties and our experiences to prepare organic–inorganic hybrid complex [35,36,37], the Co^2+^, Cu^2+^, Al^3+^, and Mn^2+^ ions were chosen and assembled with PLA1 to prepare PLA1–metal hybrid nanostructures. The Co^2+^, Cu^2+^, Al^3+^, and Mn^2+^ ions were assembled with PLA_1_ to prepare PLA_1_–metal hybrid nanostructures. The assembled hybrid nanomaterials were characterized by field emission scanning electron microscopy (SEM), Fourier infrared spectroscopy (FTIR), and *X*-ray diffraction (XRD). The enzymatic properties of the synthesized PLA_1_–metal hybrid nanostructures were further studied. This is the first study about immobilizing PLA_1_ using the organic–inorganic hybrid nanostructures method, and the results will provide a foundation and guidance for the preservation and utilization of PLA_1_.

## 2. Materials and Methods

### 2.1. Materials

The gene sequence of PLA_1_ original from *Streptomyces albidoflavus* was downloaded from the National Center for Biotechnology Information (GenBank: AB605634.1) and synthetized by GENEWIZ Biotechnology Co., Ltd. (Suzhou, China). The restriction enzymes were purchased from Thermo Fisher Scientific. Soy lecithin was purchased from Shanghai Macleans Biochemical Technology, and other required chemicals (analytical reagent grade) were supplied by local manufacturers. 

### 2.2. Preparation of PLA_1_ and Enzyme Activity Determination 

The PLA_1_ was prepared as described in our previous study [36]. 

The polyvinyl alcohol (2%) and phosphate buffer (pH 6.5) were mixed at a volume ratio of 1:1. After adding soybean lecithin (5%, *w*/*v*) into the solution, the mixture was then emulsified at 8000 rpm by a portable high-speed homogenizer named as T10 basic (IKA, Germany). The mixture (500 µL) was preheated in a 60 °C water bath for 2 min. Enzymes (10 µL) were subsequently added and incubated for another 10 min. Thereafter, 95% ethanol (2 mL) was added to terminate the enzyme reaction. The produced fatty acid was determined by using commercial free fatty acid assay kit (Nanjing Jiancheng Bioengineering Institute, Nanjing, China) followed by the manufacture’s instruction. The standard curve of free fatty acid was established at 546 nm by using a microplate reader to calculate the amount of the produced free fatty acid. One unit of enzyme activity was defined as the amount of enzyme required to produce 1 µmol of fatty acid per minute. The data were expressed as mean ± standard deviation (SD).

### 2.3. Preparation of PLA_1_–Metal Hybrid Nanostructures

PLA_1_ (0.05 mg·mL^−1^, 0.1 mg·mL^−1^, and 0.15 mg·mL^−1^) were mixed with different volumes of CoSO_4_, CuSO_4_, Al_2_(SO_4_)_3_, and MnSO_4_ in phosphate buffer (PBS, 20mM, pH 7.0) at a final concentration of 120 mM. By adjusting the volume of PBS, a final concentration of Co^2+^, Al^3+^, and Mn^2+^ metal ions at 1 mM, 2 mM, and 3 mM were achieved, respectively. For Cu^2+^, the final concentration was adjusted as 1 mM, 2 mM, 3 mM, 5 mM, 7 mM, and 9 mM. The total volume of the current immobilization system was 1 mL. After a mix and incubation at 4 °C for 24 h, 48 h, or 72 h, the above solutions were centrifugated at 12,000× *g* for 30 min to collect the precipitates. Following a wash with 20 mM PB buffer for three times, the precipitates were freeze-dried in a refrigerator at −20 °C for 3 h.

### 2.4. The Encapsulation Yield of PLA_1_

After centrifugation, the Coomassie brilliant blue method was used to measure the concentration of protein at 595 nm with a microplate reader. Bovine serum albumin (BSA) was used as the standard to determine the enzyme content in the supernatant after immobilization. The encapsulation yield (EY) is defined as the ratio of the immobilized enzyme mass to the added free enzyme mass for the characterization of the immobilization efficacy:

(1) $$\mathrm{EY}\left(\%\right)=\frac{C_{0}-C_{1}}{C_{0}}\times 100\%$$

where EY, C_0_, and C_1_ represent the encapsulation yield, total amount of added free enzyme, and the amount of free enzyme in the supernatant, respectively.

### 2.5. Field Emission Electron Microscope (FE-SEM)

The lyophilized samples with Mn^2+^, Cu^2+^, Al^3+^, and Co^2+^ were placed on the silicon wafer, gold sputter coated, and imaged by a FE-SEM (Hitachi SU8010, Tokyo, Japan). The scanning voltage was 20 kV, and the acceleration voltage was 3 kV. The contrast, brightness, and magnification were tuned to facilitate observation of sample morphology and particle size distribution with the FE-SEM.

### 2.6. Fourier Transform Infrared Spectroscopy (FTIR) Analysis

The immobilized enzyme samples were analyzed by using a Thermo Scientific Nicolet iS5 Fourier Transform Infrared Spectrometer. The lyophilized samples were ground with KBr particles to prepare transparent thin slices before inserting into the chamber of FTIR instrument. The sample spectra were collected and analyzed after collecting the reference background spectrum.

### 2.7. X-ray Diffraction (XRD) Analysis

An *X*-ray diffraction (XRD) of Rigaku SmartLab 9 kw was used to analyze the immobilized samples. About 30 mg of lyophilized samples were prepared for testing. The sample was dispersed evenly in the sample holder and was compressed with a glass plate to make the sample surface at the same level with the glass surface. The sample holder was placed in the goniometer starting from 20° and ending at 80° with increasing pressure every 30 s from 5 kV to 40 kV. The scan range was set to 5–90°, and the scan step scale was 0.2. The MDI Jade 6.0 PDF card standard library was used to compare and analyze the collected data.

### 2.8. Enzymatic Properties of PLA_1_–Metal Hybrid Nanostructures 

The enzymatic and material properties of the immobilized enzymes, including the enzymatic stability, substrate specificity, organic solvents tolerance, and thermal characteristics, were determined within the current study. 

The enzymatic stability was studied by incubating the immobilized enzymes at different temperatures (4, 25, 30, 35, 40, 45, 50, 55, and 60 °C) for 30 min and within pH buffer ranging from 4–10 at 30 °C for 1 h. After incubation, the precipitate was collected by centrifugation at 12,000× *g* for 30 min. Before detecting the enzymatic activity, the hybrid nanostructures were washed with deionized water. According to the enzyme activity detection method provided in Section 2.2, the activity of PLA_1_–metal hybrid nanostructures was measured. With blank controls of CoSO_4_, CuSO_4_, Al_2_(SO_4_)_3_, and MnSO_4_, the relative activity under different temperature was calculated by defining the enzyme activity of immobilized enzymes incubated at 4 °C as 100%. For pH stability, the highest enzyme activity measured in the experiment was considered as 100%, and the relative activity under other pH conditions were calculated accordingly.

Differential scanning calorimetry (DSC) was performed for the thermal analysis of different metal immobilized nanostructures. The sample was detected as previously described [37]. The DSC curve was drawn by setting the rate of sample absorbing or releasing heat as the ordinate and the temperature change as the abscissa by Origin software.

To investigate the storage stability, the immobilized enzymes were stored at 4 °C in a refrigerator and the enzyme activity was detected every two days as described in Section 2.2. The stability was revealed by relative activity (set the initial activity measured at the first day as 100%). 

The substrate specificity of the studied immobilized enzymes was further determined by having the enzymes react with different substrate solutions. After the reaction with 30 mM dipalmitoyl phosphatidylcholine (DPPC), dipalmitoyl phosphatidic acid (DPPA), dipalmitoyl phosphatidylethanolamine (DPPE), dipalmitoyl phosphatidylglycerol (DPPG), and dipalmitoyl phosphatidylserine (DPPS) at 35 °C for 10 min, the enzyme activity against different substrates was measured following the method described in Section 2.2. 

The tolerance of free PLA_1_ and PLA_1_–metal hybrid nanostructures to eight organic solvents (methanol, ethanol, DMSO, dioxane, tert-butanol, acetone, 2-propanol, and n-hexane) was studied. Free PLA_1_ and immobilized enzymes were immersed in an organic solvent/water (20:80, *v*/*v*) reaction system for 24 h, and the activity was measured at 12 h and 24 h as described above in Section 2.2, and displayed by relative activity (set the initial activity as 100%).

### 2.9. Statistical Analysis

The software SPSS 25.0 (SPSS Inc., Chicago, IL, USA) was employed to analyze all data. One-way ANOVA followed by LSD test was performed to evaluate the significant differences between groups. Unless stated, the results were presented by mean ± SD. 

## 3. Results and Discussion

### 3.1. Preparation of PLA_1_–Metal Hybrid Nanostructures

In this study, PLA_1_ was expressed in *Escherichia coli* BL21 (DE3) and purified by affinity purification to obtain pure PLA_1_ enzyme as one of the raw materials for preparing hybrid nanostructures [36]. To synthesize hybrid nanometer, three main steps including the formation of nucleus, the growth of metal nanostructures, and the completion of metal nanostructure self-assembly were involved. Among them, the formation of the nucleus, which would be affected by the coordination of metal phosphate and the enzymes to metal ions ratio, is the determining factor for ensuring the efficacy of immobilization [38]. Namely, parameters related to the process of immobilization would all affect the immobilization performance [39]. Therefore, taking the effects brought by buffer pH, ion concentration, and enzyme concentration into account, the immobilization conditions in this study were optimized for the preparation of PLA_1_–metal hybrid nanostructures. From the prospect of maximizing protein stability, neutral pH (7.0) was selected for the buffer solution herein as protein denaturation might occur under high acidic or basic conditions. Data shown in Table 1, Table 2, Table 3 and Table 4 detailed the parameters optimized for preparing PLA_1_–Co, PLA_1_–Cu, PLA_1_–Mn, and PLA_1_–Al, respectively. 

For PLA_1_–Co (Table 1), the highest EY and specific enzyme activity were obtained simultaneously by having 0.05 mg/mL PLA_1_ immobilized with 1 mmol/L Co^2+^. Thus, the above condition was chosen for preparing PLA_1_–Co. Table 2 shows that when the free PLA_1_ was 0.05 mg/mL, the EY of PLA_1_–Cu was 100% whilst the specific enzyme activity was below 1 U/mg, indicating that higher concentration of Cu^2+^ should be added for immobilization. To ensure good performance at both specific enzyme activity and EY, 0.15 mg/mL PLA_1_ and 7 mmol/L Cu^2+^ were then selected to construct the PLA_1_–Cu. At such conditions, the EY and specific enzyme activity of the immobilized enzyme could achieve 69% and 2.59 U/mg, respectively. From Table 3, it could be found that the EY decreased with the increase of enzyme concentration when keeping the concentration of Mn^2+^ the same. This observation indicated that free PLA_1_ could not be immobilized effectively when Mn^2+^ was insufficient. On the other hand, although the EY decreased with the increase of Mn^2+^ concentration from 1 mmol/L to 2 mmol/L^−1^ when free PLA_1_ was 0.05 mg/mL, the specific enzyme activity increased from 0.48 U/mg to 1.23 U/mg. This might be caused by the increase of the material specific area to provide more contact site for the substrate. In addition, with only 18% of the EY, the highest specific enzyme activity was achieved by complexes formed by 0.15 mg/mL enzyme and 1 mmol/L Mn^2+^. In comparison among all the studied condition, 0.05 mg/mL PLA_1_ and 2 mmol/L Mn^2+^ were selected to prepare PLA_1_–Mn with a measurement of EY and specific enzyme activity of 72% and 1.23 U/mg. The results of Table 4 revealed that the immobilized enzyme prepared by 1 mmol/L Al^3+^ and 0.05 mg/mL PLA_1_ obtained the highest EY. With the increase of Al^3+^ concentration, the EY decreased continuously. Both the EY and specific enzyme activity were low at 3 mmol/L Al^3+^, which might be because the precipitation rate was too fast to have PLA_1_ coordinated sufficiently with the Al^3+^ ions. Overall, the PLA_1_–Al hybrid nanostructures were prepared by the 0.05 mg/mL PLA_1_ and 2 mmol/L Al^3+^, under which the EY and the specific enzyme activity was 82% and 1.23 U/mg. 

### 3.2. Characterization of Metal–PLA_1_ Hybrid Nanostructures

**Figure 1:**
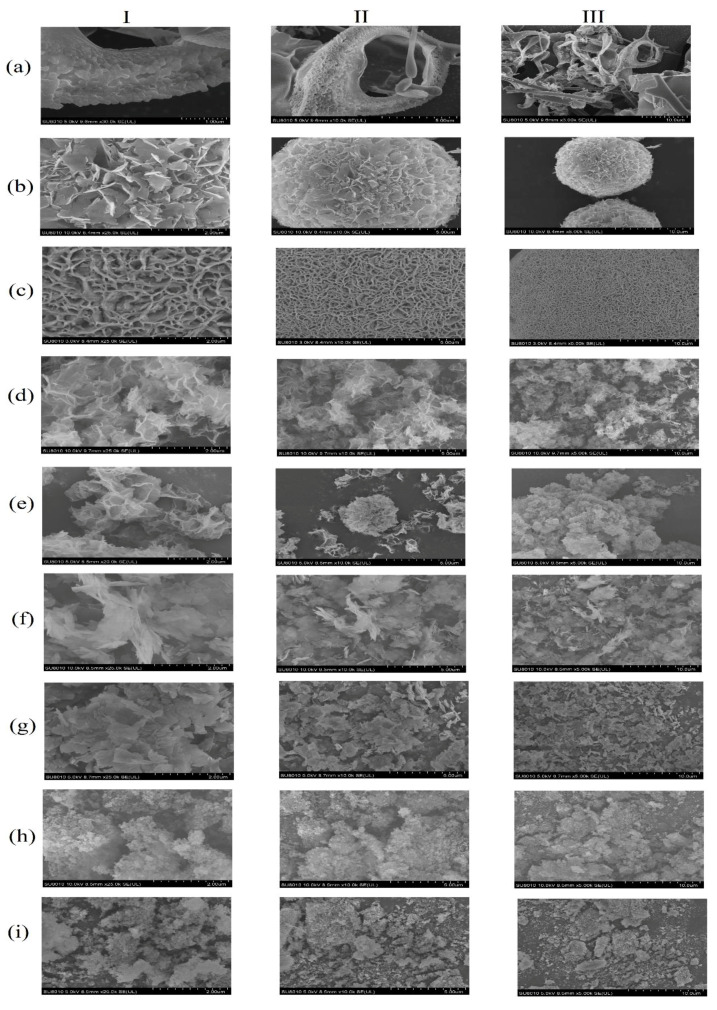
SEM images showing the hybrid metal nanostructure. (**a**) Lyophilized powder of phospholipase A_1_; (**b**) Cu_3_(PO_4_)_2_·3H_2_O; (**c**) Nanostructure of 7 mM Cu^2+^ incubated with 0.15 mg/mL PLA_1_; (**d**) CoHPO_4_·3H_2_O; (**e**) Nanostructure of 1 mM Co^2+^ incubated with 0.05 mg/mL PLA_1_; (**f**) Mn_3_(PO_4_)_2_·3H_2_O; (**g**) Nanostructure of 2 mM Mn^2+^ incubated with 0.05 mg/mL PLA_1_; (**h**) AlPO_4_; (**i**) Nanostructure of 2 mM Al^3+^ incubated with 0.05 mg/mL PLA_1_. I, II, III represented different magnification (2 µm, 5 µm, and 10 µm, respectively).

The hybrid nanostructure are the aggregation of proteins and metal ions during the synthesis process. These complexes would grow in the aggregates and undergo coordination reactions. In this study, the prepared metal–PLA_1_ were observed to form a spherical or granule shape based on the FE-SEM images (Figure 1). Figure 1a showed the SEM image of a blank control with an irregular distribution. Figure 1c,e represented enzyme nanostructures with Cu^2+^ and Co^2+^ ions, respectively, and the structure of the resulting nanomaterials could be clearly observed. In Figure 1g, the Mn^2+^ aggregated appear as petals of nanostructures, which was consistent with the literature [24]. Figure 1i represented the enzyme complexed with Al^3+^ ions, showing tiny spherical shapes. Rather than forming in a uniform structure, each individual ion complexing with the enzyme leads to a unique shape, which may be attributed to the difference in binding forces between different ions and proteins [37]. Additionally, in consideration of the amount of free enzyme applied for immobilization, the aggregation of protein and metal ions might be inhibited to some extent, and hence result in a limitation on the growth of nanostructures [40].

**Figure 2:**
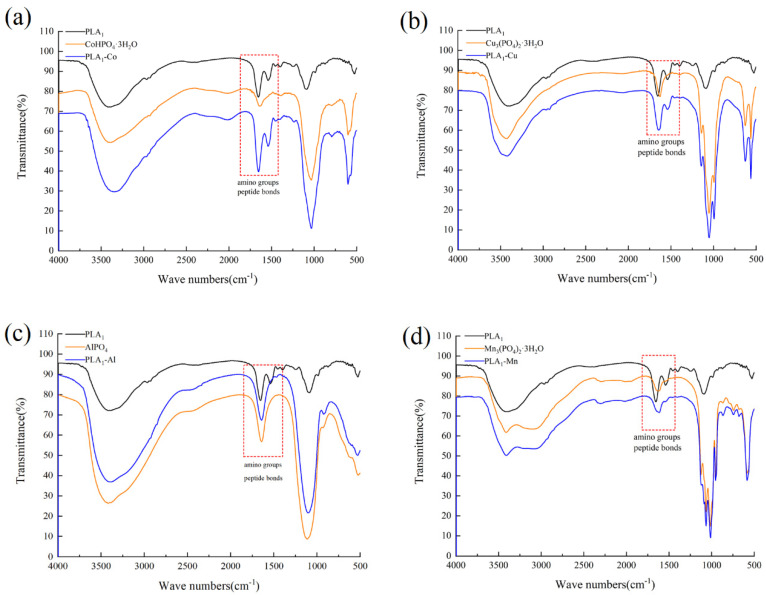
FTIR analysis of immobilized enzymes. (**a**) PLA_1_–Co hybrid nanostructures. (**b**) PLA_1_–Cu hybrid nanostructures. (**c**) PLA_1_–Al nanostructures. (**d**) PLA_1_–Mn hybrid nanostructures. The spectra shows typical peaks of free enzymes and related metal materials, and the peaks corresponding to amino groups (1600 cm^−1^) and peptide bonds (1645 cm^−1^) are marked with rectangles.

FTIR analysis shown in Figure 2 indicated that the vibration frequency of each hybrid nanostructure was different. However, it should be noted that the results showed a peak curve at 500 cm^−1^ and 1000 cm^−1^ for all hybrid nanostructures. 500 cm^−1^ is the P=O bending vibration peak, and 1000 cm^−1^ represents the P-O stretching vibration peak [40,41]. Based on current observations, all the prepared hybrid nanostructures were characterized with the composition of phosphate groups. In addition, the infrared spectra of PLA_1_–Co, PLA_1_–Cu, PLA_1_–Al, and PLA_1_–Mn showed typical peaks of 1600 cm^−1^ amino group and 1645 cm^−1^ peptide bonds, indicating the formation of amide, carboxyl, and hydroxyl groups with different metal ions [37]. The results demonstrated that the hybridization of the nanostructure structure was successfully formed.

**Figure 3:**
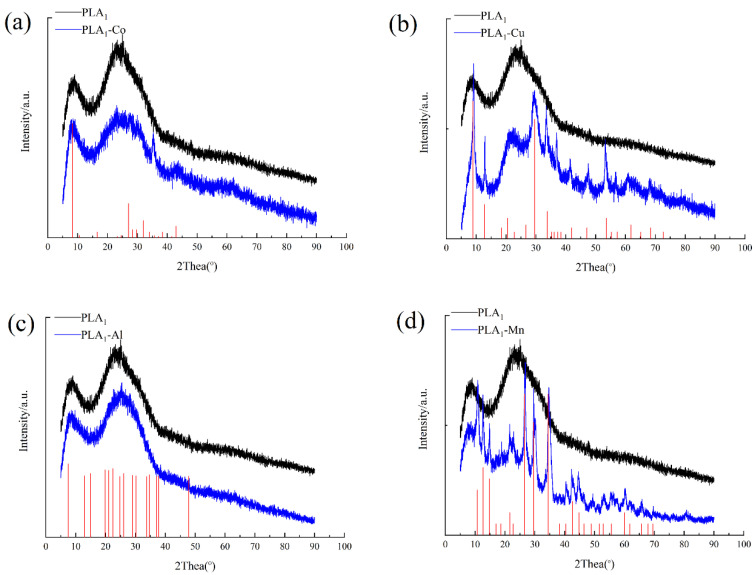
XRD analysis of immobilized enzymes. (**a**) PLA_1_–Co black line, PLA_1_–Co hybrid nanostructures blue line, and XRD pattern of CoHPO_4_·3H_2_O (CPDS, card 00-39-0702). (**b**) PLA_1_–Cu black line, PLA_1_–Cu hybrid nanostructures blue line, and XRD pattern of Cu_3_(PO_4_)_2_·3H_2_O (CPDS, card 00-22-0548). (**c**) PLA_1_–Al black line, PLA_1_–Al hybrid nanostructures blue line, and XRD pattern of AlPO_4_ (CPDS, card 00-41-0044). (**d**) PLA_1_–Mn black line, PLA_1_–Mn hybrid nanostructures blue line, and XRD pattern of Mn_3_(PO_4_)_2_·3H_2_O (CPDS, card 00-03-0426).

XRD illustrates a physical image analysis of substances. In order to verify the crystal formation of metal–PLA_1_ hybrid nanostructures, the XRD was employed to analyze the powders of PLA_1_ and these four kinds of PLA_1_–metal hybrid nanostructures. The X-ray diffraction peaks of PLA_1_–Co, PLA_1_–Cu, PLA_1_–Al, and PLA_1_–Mn hybrid nanostructures could be obtained when comparing the results with the standard card (CPDS, card 00-39-0702), (CPDS, card 00-22-0548), (CPDS, card 00-41-0044), and (CPDS, card 00-03-0426). Generally, the peaks recognized from samples showed nearly identical patterns to the standard card, indicating that the metal hybrid nanostructures have good crystallinity. The diffraction peaks of each metal ion in the corresponding hybrid nanostructures were different, perhaps because of the unique crystalline structures as shown in Figure 3.

### 3.3. Analysis of the Properties of Immobilized Nanostructures

The stability of immobilized enzymes is the key factor for application in the industry. Previous studies have indicated that the hybrid nanostructures formatted by metal ions with enzyme could significantly enhance the stability of enzymes [36,38]. Herein, the tolerance of four kinds of PLA_1_–metal hybrid nanostructures to temperature, pH, long-term storage, and organic solvent was investigated to estimate the stability of the immobilized enzyme.

**Figure 4:**
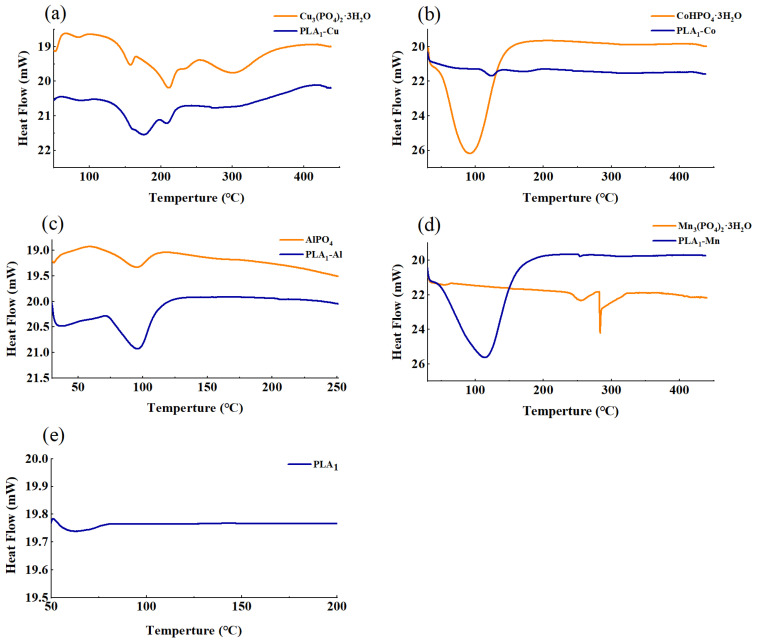
DSC analysis of metal–PLA_1_ hybrid nanostructures. (**a**) The T_m_ value of Cu_3_(PO_4_)_2_·3H_2_O was 210.71 °C, and the T_m_ value of PLA_1_–Cu was 173.14 °C. (**b**) The T_m_ value of CoHPO_4_·3H_2_O was 91.09 °C, and the T_m_ value of PLA_1_–Co was 123.67 °C. (**c**) The T_m_ value of AlPO_4_was 94.72 °C, and the T_m_ value of PLA_1_–Al was 96.31 °C. (**d**) The T_m_ value of Mn_3_(PO_4_)_2_·3H_2_O was 282.03 °C, and the T_m_ value of PLA_1_–Mn was 114.79 °C. (**e**) The T_m_ value of free PLA_1_ was 65.59 °C.

In the aspect of thermostability of the original metal phosphates and immobilized enzymes, DSC was used to determine the effect of temperature on the material stability. In Figure 4, the endothermic peak could be observed as a single peak for each sample. The initial T_m_ value of the free enzyme was only 65.59 °C and increased to 123.67 °C, 173.14 °C, 96.31 °C, and 114.79 °C after being immobilized by Co^2+^, Cu^2+^, Al^3+^, and Mn^2+^, respectively, suggesting an improvement of thermal stability for PLA_1_ after immobilization. This change of property might be affected by the formation of stable crystal structure, which could enhance material resistance to temperature [42]. However, the results of DSC analysis are only applicable to PLA_1_ in solid state, and the thermal stability of the immobilized enzyme in liquid circumstances require further analysis.

**Figure 5:**
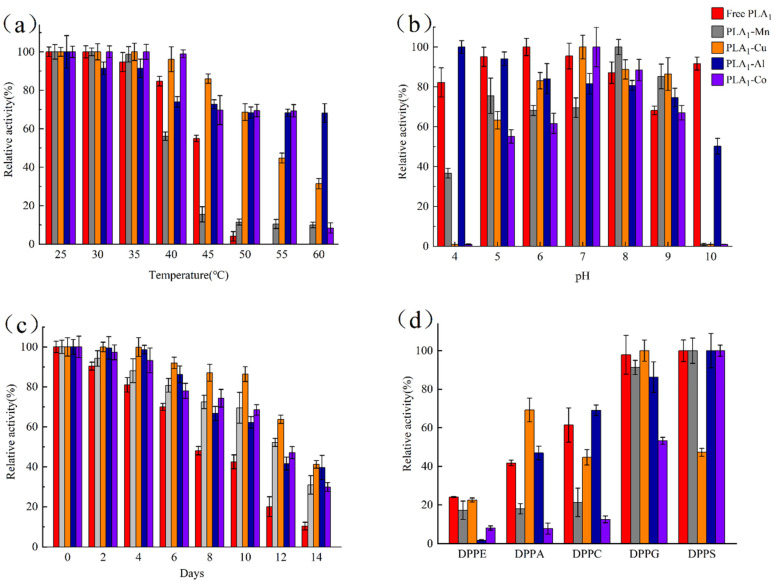
Studies on temperature (**a**), pH (**b**), storage stability (**c**), and substrate specificity (**d**) of hybrid enzyme nanostructures.

The temperature tolerance of these four immobilized enzymes, as well as the free enzyme, was further evaluated by measuring the variation of enzyme activity after a 30-min incubation against different temperature. As shown in Figure 5a, the enzymatic activity of both free and immobilized enzyme would decrease with the increase of temperature. However, the free PLA_1_ was observed to lose over 90% of the native activity after an incubation at 50 °C for 30 min and have no activity left when the temperature was 55 °C. In comparation, even for the worst tolerated immobilized enzyme (PLA_1_–Mn), about 10% of the activity could be maintained at 60 °C whilst there was only 56% and 17% activity remaining at 40 and 45 °C, which was lower than that of the free enzyme. For the other three immobilized enzymes, it was found to have better tolerance to temperature than the free PLA_1_ and around 70% of the activity remained after being incubated at 50 °C. Among these three, PLA_1_–Al hybrid nanostructures were verified to be the highest temperature-resistant. Within the range between 50 to 60 °C, almost 70% of the enzyme activity could be maintained for PLA_1_–Al, owing to the possibility that compounds composed of Al^3+^ were normally characterized with high temperature resistance [43]. It should also be noticed that although the remaining activity of both PLA_1_–Cu and PLA_1_–Co was less than that of PLA_1_–Al at 60 °C, these two immobilized enzymes still showed a relative good thermostability against other temperature. Given all that, the above results suggested that the thermal stability of PLA_1_ could be promoted via preparing the metal hybrid nanostructures.

Figure 5b shows the pH stability of the prepared immobilized enzymes by having the enzyme incubated within a different pH buffer (4–10) for 1 h. The results indicated that above 50% of the enzymatic activity of the immobilized enzyme could be maintained at a pH of 5–9. In addition, there was a variation of pH tolerance for different metal–PLA_1_ over the studied pH. Compared with free PLA_1_, Al^3+^ was found to improve the enzymatic resistance under acid conditions (pH 4), while the enzyme stability was benefited by being immobilized with Cu^2+^ when the pH was between 7 and 9. For PLA_1_–Mn and PLA_1_–Co, a higher pH-resistant was observed under alkaline (pH 8–9) and neutral condition (pH 7), respectively. In general, Al^3+^ promoted the overall pH stability of PLA_1_ most among these four metal ions. By contrast, when the pH was low (pH 4) or high (pH 10), enzyme immobilized with Cu^2+^, Co^2+^, or Mn^2+^ could not retain the initial activity. Theoretically, the organic–inorganic hybrid nanostructures are an enzyme–phosphate co-crystallized solid substance that is formatted by the coordination between the chemical bonds within the enzyme and the phosphate precipitation, which is formed with metal and phosphate ions [40]. Based on this mechanism, phosphates would precipitate when the pH is low, while the structure of nanostructures would be disrupted if the enzyme complexation with phosphate is interfered by high pH. As a result, the immobilization of enzyme could not be performed effectively, and even the immobilized enzyme would release into the solution in the form of free enzyme. In consideration of the observation in this study, the above theory might provide an explanation for why there was a loss of enzyme activity at the pH of 4 or 10 when PLA_1_ was immobilized with Cu^2+^, Co^2+^, or Mn^2+^.

To evaluate the long-term storage stability, the purified immobilized enzymes were stored at 4 °C after dialysis with PBS (20 mmol∙L^−1^, pH 7). In Figure 5c, it could be found that the storage stability of immobilized PLA_1_ was obviously higher than that of the free enzyme. After a storage of 14 days, the PLA_1_–Cu and PLA_1_–Al hybrid nanostructures could still maintain above 40% enzyme activity, whilst the free enzyme lost about 90% of the initial activity. Despite relative lower storage stability for PLA_1_–Co and PLA_1_–Mn (30% left over 14 days), the immobilization of PLA_1_ by metal ions could still be a valid method in the aspect of promoting the long-term storage stability of enzymes.

The substrate specificity of immobilized and free enzymes against DPPC, DPPS, DPPA, DPPE, and DPPG was detected herein (Figure 5d). Compared with the free enzyme, PLA_1_–Mn and PLA_1_–Al shared similar ability to hydrolyze DPPS and DPPG. However, there was an obvious decrease of activity on DPPE, DPPA, and DPPC for enzyme immobilized with Mn^2+^. Considering the possibility that the exposure of enzymes to the substrate might be restricted after immobilization [44], the above three substrates would be hard to come into contact with the enzyme as their water solubility is worse than that of DPPS and DPPG. In addition, PLA_1_–Al was found to show the worst activity on DPPE. A contribution to this phenomenon might be the formation of zwitterion, which was a stable structure produced by the reaction between Al^3+^ and the ethanolamine group within DPPE. This zwitterion would lead to the increase of hydrophobicity with the reduction in polarity of substrate, and hence resulting in the decrease of enzymatic hydrolysis ability on DPPE. For PLA_1_–Co, a comparable activity was only observed on DPPS. As there is an existence of serine group within DPPS, a specific function between Co^2+^ and serine might occur, thus shortening the distance between the substrate and the immobilized enzyme. Meanwhile, since DPPS is characterized with relatively good water solubility, it will be easier to hydrolyze by PLA_1_–Co. When immobilized the enzyme with Cu^2+^, the specificity was found to associate with the charge on substrates. Poorer hydrolysis capability was identified against choline charged DPPC and serine charged DPPS, whilst a better activity on the uncharged DPPA was observed. The above result suggested that there might be a rejection of PLA_1_–Cu against phospholipids substrate with charged polar head. Consequently, it would be difficult for the substrate to bond with the immobilized enzyme.

**Figure 6:**
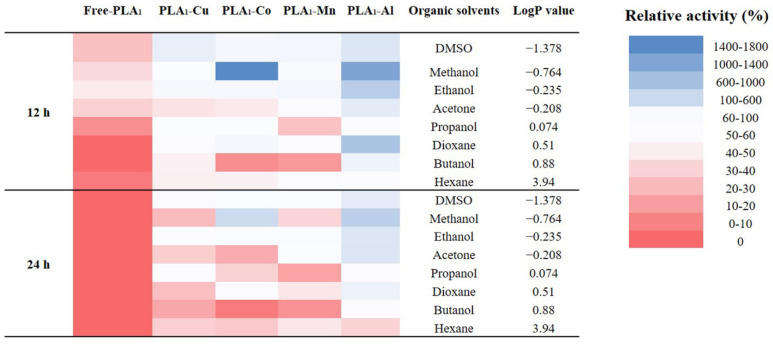
The tolerance of immobilized enzyme to eight kinds of organic solvents.

The tolerance of immobilized enzyme to organic solvents was determined against eight common organics with a varied value of LogP. It could be found that both the free and immobilized enzyme showed a better resistance to hydrophilic solvents than to the hydrophobic ones (Figure 6). This might be related to the natural enzymatic property of PLA_1_, which could maintain above 40% of the activity after an incubation with hydrophilic solvents for 12 h. For comparison, the free enzyme only revealed less than 20% of the initial activity when incubated in hydrophobic solvents. Regardless of the applied metal phosphates, the tolerance to organic solvents was significantly improved, suggesting a stable structure formed between the enzyme and metal phosphates. Meanwhile, it might attribute the high relative enzymatic activity to the increased contact aera between substrate and enzyme resulting from the formation of metal hybrid nanostructures. Regarding different metal ions, Al^3+^ showed the best effect on promoting enzymatic organic solvents tolerance. In association with the SEM results (Figure 1), the morphological characteristics of PLA_1_–Al might relate to current observations. Unlike the nanostructures (PLA_1_–Cu and PLA_1_–Co) or the lamellar structure (PLA_1_–Mn), PLA_1_–Al was formed as the grainy structure. With this compact structure, the immobilized enzyme could be well prevented from the influence introduced from organic solvents.

## 4. Conclusions

In this study, the free PLA1 was immobilized by assembling organic–inorganic hybrid nanostructures with four kinds of metal ions. FE-SEM, FTIR, XRD, and DSC analysis showed that PLA_1_–metal hybrid nanostructures was successfully formed at 4 °C after 72 h. The immobilized enzymes showed excellent temperature stability versus free PLA_1_, the Tm value of PLA_1_–Co, PLA_1_–Cu, PLA_1_–Al, and PLA_1_–Mn as measured by DSC had increased from 65.59 °C to 123.67 °C, 173.14 °C, 96.31 °C, and 114.79 °C, respectively. This study offered an efficient immobilization method for PLA_1_ and provides a basis for the industrial application of PLA_1_.

## Figures and Tables

**Table 1:** Preparation of PLA_1_–Co hybrid nanostructures.

EC (mg/mL)	Co^2+^ (mM)	24 h		48 h		72 h	
		EY %	SEA (U/mg)	EY %	SEA (U/mg)	EY %	SEA (U/mg)
0.05	1	84 ± 1	6.84 ± 0.05	100 ± 3	4.41 ± 0.08	100 ± 9	4.80 ± 0.07
0.10	1	46 ± 6	3.05 ± 0.04	99 ± 4	4.99 ± 0.01	83 ± 7	4.50 ± 0.04
0.15	1	24 ± 5	7.47 ± 0.15	82 ± 5	3.98 ± 0.06	88 ± 8	3.88 ± 0.02
0.05	2	100 ± 1	1.58 ± 0.12	100 ± 7	1.32 ± 0.01	99 ± 5	2.24 ± 0.03
0.10	2	89 ± 7	4.72 ± 0.36	100 ± 1	0.79 ± 0.01	28 ± 16	2.19 ± 0.04
0.15	2	68 ± 3	6.30 ± 0.25	92 ± 7	1.05 ± 0.01	74 ± 10	1.63 ± 0.05
0.05	3	100 ± 2	0.53 ± 0.01	100 ± 2	0.42 ± 0.01	82 ± 9	1.49 ± 0.01
0.10	3	85 ± 2	2.60 ± 0.06	100 ± 1	0.45 ± 0.01	78 ± 1	4.07 ± 0.07
0.15	3	73 ± 1	1.08 ± 0.01	98 ± 11	0.37 ± 0.04	38 ± 1	1.65 ± 0.02

EC: enzyme concentration; EY: encapsulate yield; SEA: specific enzyme activity.

**Table 2:** Preparation of PLA_1_–Cu hybrid nanostructures.

EC (mg/mL)	Cu^2+^ (mM)	24 h		48 h		72 h	
		EY %	SEA (U/mg)	EY %	SEA (U/mg)	EY %	SEA (U/mg)
0.05	1	100 ± 6	1.80 ± 0.08	100 ± 1	0.93 ± 0.01	100 ± 2	0.88 ± 0.02
0.1	1	98 ± 2	1.33 ± 0.03	99 ± 1	1.05 ± 0.01	74 ± 4	0.72 ± 0.03
0.15	1	62 ± 7	1.55 ± 0.17	68 ± 8	1.34 ± 0.15	55 ± 1	0.85 ± 0.02
0.05	2	100 ± 1	1.14 ± 0.01	100 ± 6	1.42 ± 0.03	100 ± 1	0.86 ± 0.01
0.1	2	91 ± 4	1.44 ± 0.06	100 ± 0	0.65 ± 0.04	78 ± 1	1.19 ± 0.01
0.15	2	67 ± 1	1.26 ± 0.02	80 ± 9	0.69 ± 0.08	76 ± 10	0.70 ± 0.09
0.05	3	100 ± 4	0.80 ± 0.02	100 ± 3	0.57 ± 0.01	100 ± 1	0.46 ± 0.01
0.1	3	99 ± 1	0.78 ± 0.01	100 ± 1	0.52 ± 0.01	90 ± 4	0.30 ± 0.01
0.15	3	86 ± 1	0.95 ± 0.01	83 ± 2	0.58 ± 0.02	72 ± 3	0.38 ± 0.01
0.05	5	90 ± 10	0.58 ± 0.04	100 ± 1	0.43 ± 0.01	100 ± 1	0.58 ± 0.01
0.1	5	51 ± 1	0.69 ± 0.08	46 ± 4	0.59 ± 0.06	48 ± 6	1.34 ± 0.02
0.15	5	44 ± 1	0.89 ± 0.01	47 ± 1	0.07 ± 0.01	49 ± 2	2.27 ± 0.03
0.05	7	100 ± 1	2.48 ± 0.07	100 ± 1	2.24 ± 0.01	100 ± 1	1.22 ± 0.05
0.1	7	46 ± 1	1.51 ± 0.05	81 ± 6	2.31 ± 0.05	81 ± 4	1.63 ± 0.03
0.15	7	32 ± 1	1.37 ± 0.04	34 ± 1	1.26 ± 0.01	69 ± 1	2.59 ± 0.07
0.05	9	100 ± 1	2.47 ± 0.07	100 ± 1	1.88 ± 0.01	100 ± 1	0.74 ± 0.07
0.1	9	57 ± 3	0.23 ± 0.01	81 ± 5	2.36 ± 0.01	81 ± 1	2.15 ± 0.04
0.15	9	51 ± 1	2.15 ± 0.01	42 ± 2	2.31 ± 0.03	46 ± 1	2.23 ± 0.02

EC: enzyme concentration; EY: encapsulate yield; SEA: specific enzyme activity.

**Table 3:** Preparation of PLA_1_–Mn hybrid nanostructures.

EC (mg/mL)	Mn^2+^ (mM)	24 h		48 h		72 h	
		EY %	SEA (U/mg)	EY %	SEA (U/mg)	EY %	SEA (U/mg)
0.05	1	92 ± 7	0.64 ± 0.05	100 ± 15	0.57 ± 0.07	92 ± 5	0.48 ± 0.03
0.1	1	43 ± 10	1.11 ± 0.02	57 ± 1	0.77 ± 0.01	30 ± 4	1.09 ± 0.14
0.15	1	29 ± 1	1.48 ± 0.07	29 ± 5	0.96 ± 0.08	18 ± 2	1.60 ± 0.20
0.05	2	74 ± 1	1.17 ± 0.02	100 ± 18	0.76 ± 0.05	72 ± 5	1.23 ± 0.08
0.1	2	27 ± 1	2.04 ± 0.07	45 ± 3	0.80 ± 0.05	38 ± 1	1.30 ± 0.03
0.15	2	15 ± 1	2.22 ± 0.02	29 ± 2	0.97 ± 0.05	24 ± 1	1.56 ± 0.07
0.05	3	62 ± 10	3.00 ± 0.02	100 ± 2	0.66 ± 0.01	63 ± 4	0.85 ± 0.05
0.1	3	28 ± 1	1.43 ± 0.03	48 ± 3	0.69 ± 0.05	32 ± 5	0.90 ± 0.14
0.15	3	8 ± 2	5.58 ± 0.04	32 ± 1	0.83 ± 0.02	17 ± 0	1.13 ± 0.01

EC: enzyme concentration; EY: encapsulate yield; SEA: specific enzyme activity.

**Table 4:** Preparation of PLA_1_–Al hybrid nanostructures.

EC (mg/mL)	Al^3+^ (mM)	24 h		48 h		72 h	
		EY %	SEA (U/mg)	EY %	SEA (U/mg)	EY %	SEA (U/mg)
0.05	1	89 ± 7	0.61 ± 0.04	100 ± 21	0.38 ± 0.03	95 ± 2	0.50 ± 0.01
0.10	1	20 ± 3	1.01 ± 0.13	42 ± 6	0.67 ± 0.03	97 ± 6	0.37 ± 0.04
0.15	1	12 ± 1	1.16 ± 0.09	49 ± 24	0.90 ± 0.07	27 ± 3	0.94 ± 0.12
0.05	2	71 ± 2	1.20 ± 0.03	99 ± 16	0.45 ± 0.01	82 ± 1	0.79 ± 0.01
0.10	2	28 ± 3	1.19 ± 0.14	53 ± 4	0.40 ± 0.08	66 ± 1	0.61 ± 0.01
0.15	2	13 ± 1	1.95 ± 0.10	52 ± 12	0.35 ± 0.04	53 ± 1	0.64 ± 0.01
0.05	3	46 ± 5	1.36 ± 0.15	83 ± 10	0.54 ± 0.05	74 ± 1	0.55 ± 0.01
0.10	3	17 ± 0	1.80 ± 0.02	40 ± 4	0.62 ± 0.01	28 ± 1	0.88 ± 0.01
0.15	3	3 ± 3	1.17 ± 0.02	21 ± 7	0.84 ± 0.04	25 ± 1	1.07 ± 0.01

EC: enzyme concentration; EY: encapsulate yield; SEA: specific enzyme activity.

## Data Availability

The data presented in this study are available on request from the corresponding author.
